# Cross-Cultural Perspectives on Insect-Based Foods: Insights from Consumers in Greece and Ireland

**DOI:** 10.3390/foods14030490

**Published:** 2025-02-03

**Authors:** Leocardia Ranga, Malamatenia Panagiotou, Francesco Noci, Maria Charalampidou, Konstantinos Gkatzionis, Maria Dermiki

**Affiliations:** 1 Department of Health and Nutritional Sciences, Faculty of Science, Atlantic Technological University, F91 YW50 Sligo, Ireland; leocardia.ranga@research.atu.ie; 2 Laboratory of Consumer and Sensory Perception of Foods & Beverages, Department of Food Science and Nutrition, University of the Aegean, Metropolite loakeim 2, 81400 Myrina, Greece; teniapanag@aegean.gr (M.P.); fns20156@fns.aegean.gr (M.C.); kgkatzionis@aegean.gr (K.G.); 3 Department of Sports, Exercise and Nutrition, School of Science and Computing, Atlantic Technological University, H91 T8NW Galway City, Ireland; francesco.noci@atu.ie

**Keywords:** entomophagy, novel food, sustainable alternatives, alternative protein, purchasing intent, food choice

## Abstract

In the context of globalization, cross-cultural studies have become increasingly important for understanding differences in consumer acceptance of various foods. This study examines and compares the acceptance of insect-based foods between consumers in Greece and Ireland, two EU countries where insect-based foods are not widely available. An online survey was distributed in both countries and responses from 489 participants (Greece: *n* = 283; Ireland: n = 206) were analysed, using non-parametric tests for the quantitative data, and a combination of thematic and content analysis for the qualitative data. Overall, the Mann–Whitney U test showed that participants from Greece were significantly less willing to consume insect-based foods than those in Ireland. Among EU-approved insects, the Friedman test showed that participants in Ireland significantly preferred yellow mealworms over house crickets and migratory locusts, whereas participants in Greece showed no significant preference among these species. Both groups were more willing to consume insect-based foods when the insects were not visible, while they differed in their preference of inclusion percentage of insect protein in foods. However, no differences were found in the willingness to consume different types of non-visible insect products. The Mann–Whitney U test showed that participants in Ireland could be more influenced to consume insect-based foods by external factors, with live demonstrations by chefs being the most influential. However, family members would be the most influencing factor for those from Greece. Nuances in participants’ willingness to buy insect-based foods are presented and discussed. These findings could inform strategies aimed at increasing the acceptance of insects as food among consumers in European countries with limited exposure to such products.

## 1. Introduction

The growing world population has led to increased demand for animal protein [1,2]. Meeting this demand through the expansion of animal production exacerbates environmental challenges while further straining animal welfare. This underscores the urgent need for sustainable alternatives [3,4]. Insects have emerged as a promising alternative, as highlighted in several studies [5,6,7,8]. Life cycle assessments have shown that insects generate significantly lower GHG emissions than poultry or livestock for the same amount of protein produced [6,9,10]. While their nutritional composition varies, insects generally contain comparable protein levels and higher polyunsaturated fatty acids than traditional animal products [11].

For centuries, insects have formed part of the culinary tradition of consumers in several regions, including Africa, Eastern Asia, and South America [12]. In other countries which did not have this tradition, the acceptance of insects as food has been and continues to be explored. However, this exploration has progressed unevenly across these countries. In their review published in 2022, Kröger et al. [13] noted significant disparities in the extent of research conducted in Western countries. For instance, some countries showed relatively advanced efforts with over twenty studies, while others had conducted only one study each [13].

The willingness to consume and purchase insect-based foods, along with the factors influencing these decisions, varies significantly across countries [14]. This highlights the importance of continued efforts in exploring consumer acceptance of these foods in countries with limited information on this aspect [15]. These variations are particularly evident in cross-cultural studies [16]. In an increasingly globalised world, an understanding of the cross-cultural differences in consumer perceptions of different foods is essential [17].

Studies have been conducted comparing consumer acceptability of insect-based foods between countries with a traditional entomophagy culture and those without [14,16,18,19,20,21,22,23]. The former were generally confirmed to be more accepting of insects as food than the latter [16,18,19,20,21]. Indeed, familiarity with insects as food is one of the strongest motivating factors for acceptance [24,25,26,27,28].

Despite cultural factors associated with entomophagy traditions or lack thereof, differences have been found to exist between consumers in countries without such traditions, such as Western countries.

In the EU, insects need to be authorised under the novel food regulations before they can be placed on the market for human consumption [29]. To date, the European Commission has authorised *Tenebrio molitor* larva (yellow mealworms), *Acheta domesticus* (house crickets), *Locusta migratoria* (migratory locusts), and *Alphitobius diaperionus* larva (lesser mealworms) as novel foods in the EU [30,31,32,33]. These regulations apply to all member states in the EU which potentially facilitate cross-cultural comparisons of entomophagy acceptance between different member states.

While cross-cultural comparisons on the acceptance of insects as food have been conducted between several countries without entomophagy traditions, including EU countries, there are some gaps in the available literature. For example, although there is limited information on the acceptance of insect-based foods among consumers in Greece [34,35,36] and Ireland [37,38,39], there are no cross-cultural comparisons that have been conducted between these two EU countries to date. One is located in the southeast (Greece) and another in the northwest (Ireland) of Europe and both have different culinary traditions, two contributing factors (regional location and food culture) to differences in insect-based food acceptance reported in past cross-cultural studies [40,41,42]. Furthermore, the recent sustainable development report shows that both Greece and Ireland are currently facing ”major challenges” towards achieving the sustainable development goals (SDG) 12 and 13 of ”responsible consumption and production” and ”climate action” [43]. Introducing insect-based food to the supply chain could potentially contribute towards existing efforts in achieving these SDGs.

The current study seeks to provide cross-cultural insights into the acceptance of insect-based foods between consumers in Greece and Ireland. Specifically, the current study aims to answer the following research questions when comparing consumers in Greece and Ireland:

- To what extent are they willing/unwilling to consume insect-based foods?
- What are the reasons for their willingness/unwillingness to consume insect-based foods?
- Which factors can potentially influence them to consume insect-based foods?
- To what extent would they be willing/unwilling to buy insect-based foods?

## 2. Materials and Methods

### 2.1. Study Design and Sampling

This study employed a pragmatic approach to address the research questions, using an embedded mixed methods design (QUAN [qual]) [44]. Insights from focus group discussions [15], along with findings from the literature review, informed the development of an online survey (QUAN [qual]). Ethical approval for the study was granted by the Institute Research Ethics Committee of Atlantic Technological University (ATU), Sligo, Ireland (Ref No. 2022016). The online survey was disseminated via the email systems of ATU in Ireland and the University of the Aegean in Greece, as well as through social media platforms (LinkedIn, Facebook, X) and notice boards in local supermarkets and other establishments. Data collection occurred between November 2023 and January 2024.

### 2.2. Survey Construction

The survey used in this study initially was developed in English and then translated into Greek by a translator and a linguist specializing in sensory and consumer research. To ensure translation accuracy, the back translation method was employed [45]. Additional adjustments were made based on feedback from pilot testing with a small group of consumers in Greece and Ireland, ensuring clarity and comprehensibility across both cultural contexts. The final version of the survey comprised four sections.

The first section included questions on participants’ ranking of food choice motives in order of personal importance, their agreement with seven items from the Food Neophobia Scale developed by Pliner and Hobden [46] (rated on a 5-point scale from “strongly disagree” to “strongly agree”), and their previous knowledge and experience with insect-based foods. The question structure relating to the participants’ food choice motives and previous experience was adapted from established methodologies in previous studies [40,47,48,49].

The second section assessed participants’ willingness to consume insect-based foods and the factors influencing this willingness. Participants were shown photos of three insect species approved as novel food in the EU (yellow mealworms, migratory locusts, and house crickets) and asked to rate their willingness to consume each species on a 5-point scale ranging from “extremely unwilling” to “extremely willing”. The photos were that of whole insect species in their dried form as could be seen in the market (see Table S1). They were also asked to indicate their level of agreement with various statements about reasons for their willingness or unwillingness to consume insect-based foods, followed by open-ended questions for additional reasons. Participants were then asked about their willingness to consume different insect-based foods where insects were visible or invisible. Thereafter, they were informed that “insects are generally used as ingredients in a number of foods because of their high protein content”. This was followed by a question regarding the percentage of substitution with insect protein in a high-protein product that participants would be willing to consume. The structure of the questions relating to willingness to consume various insect-based foods was adapted from Woolf et al. [50].

The third section focused on participants’ willingness to buy insect-based foods and the degree to which external factors influenced their willingness to consume them. Participants responded to nine statements assessing their willingness to buy, rating each on a 5-point scale from “strongly disagree” to “strongly agree”. The statements were designed to capture those who would never buy insect-based foods, those willing to try if only offered for free, and those willing to buy under specific conditions. A quality check question adopted from Lammers et al. [51] was included. Participants also indicated the extent to which peers, family, social media, television programmes, and a chef would influence their consumption of insect-based foods on a 5-point scale from “definitely will not” to “definitely will”.

The final section gathered sociodemographic information, including participants’ age, gender, farming background, and dietary habits. The questions used in the survey are presented in Table S1 in the Supplementary Materials.

### 2.3. Data Analysis

A total of 571 participants (246 from Ireland and 325 from Greece) completed the online survey. However, the responses from those participants that failed the quality control question “Select ‘somewhat disagree’ as proof that you are reading the text” [51] were excluded. Therefore, a total of 489 participants (206 from Ireland and 283 from Greece) were analysed.

The quantitative data were analysed using SPSS (IBM^®^ version 29.0.1). Descriptive statistics were applied to profile participants based on age, gender, farming background, diet, and previous knowledge or experience with insect-based foods. Chi-square tests were conducted to determine if there were significant differences between the characteristics of participants in Greece and Ireland. The Friedman test was used to rank participants’ food choice motives, willingness to consume EU-approved insect species and other insect-based foods, and external influences on their willingness to consume insect-based foods. When the Friedman test indicated significant differences, post hoc pairwise comparisons with Bonferroni correction were performed to pinpoint where these differences occurred.

The Mann–Whitney U test was used to determine significant differences between participants in Greece and Ireland in terms of food choice motives, willingness to consume and buy insect-based foods, susceptibility to external influences, and agreement with product-related reasons for willingness or unwillingness to consume insect-based foods, as well as items from the Food Neophobia Scale. Spearman correlations were used to explore the relationship between participants’ agreement with reasons for willingness or unwillingness to consume insect-based foods and their actual willingness to consume various insect-based foods. The significance threshold for all the quantitative analysis was set at *p* < 0.05.

The qualitative data underwent thematic analysis, following Braun and Clarke’s six phase framework [52,53]. Content analysis was then used to quantify the codes and subthemes generated from the thematic analysis according to their frequency [54].

## 3. Results

### 3.1. Profile of the Participants

There were no significant differences (*p* > 0.05) between the distribution of participants in Greece and Ireland across age groups, gender, diet, prior knowledge of insect-based foods, or consumption experience (see Table 1). While less than half of the participants in both countries had a farming background, this was significantly (*p* < 0.001) higher in Ireland (29.6%) than in Greece (14.5). Over 90% of participants in both countries had heard of insect-based foods before, but a significantly higher proportion (*p* < 0.001) in Ireland (31.6%) had previously consumed them compared to Greece (6.7%) (see Table 1).

Table 2 highlights the most and least important food choice motives for participants in Greece and Ireland. The Friedman ranking of these motives within each country was statistically significant (*p* < 0.001). In both countries, health was the most important, followed by pleasurable sensory experiences. When comparing the two, cultural motives such as “it fits in with my culture” and “it is like the one I ate when I was a child” were significantly more important for participants in Greece (*p* < 0.001). Conversely, affordability, variety of different ingredients, and environmental friendliness were more important to participants in Ireland (*p* < 0.001). However, environmental impact was ranked as the third least important motive in Ireland and the least important in Greece (see Table 2).

The level of agreement with the Food Neophobia Scale items (mean ± standard deviation) is presented in Table S2. Participants in Greece showed significantly higher food neophobia, with higher scores on items such as “I don’t trust new foods” (*p* < 0.001), “I like foods from different cultures” (reverse scored; *p* < 0.001), “At dinner parties, I will try new foods” (reverse scored; *p* = 008), “I am afraid to eat things I have never seen before” (*p* = 0.007) and “I will eat almost anything” (reverse scored; *p* < 0.001).

### 3.2. Willingness to Consume Insect-Based Foods

The willingness to consume various insect-based foods differed significantly between participants in Greece and Ireland (*p* < 0.001). Participants in Ireland were notably more willing to consume all types of insect-based foods explored in this study compared to those in Greece (see Table 3). In Greece, the percentage of participants who were “extremely unwilling” to consume insect-based foods ranged from 51% to 79%, whereas, in Ireland, this ranged from 20% to 46%, depending on the food. Conversely, those “extremely willing” to consume insect-based foods ranged from 0% to 5% in Greece and from 1% to 26% in Ireland. When combining the “extremely willing” and “somewhat willing” categories, the proportion of participants willing to consume insect-based foods ranged from 2% to 24% in Greece and from 13% to 64% in Ireland, depending on the food (see Table 3).

Table 4 presents the ranking of EU-approved insect species and insect-containing foods based on participants’ willingness to consume them. Among the EU-approved insects, participants in both Greece and Ireland were most willing to consume yellow mealworms, followed by house crickets and migratory locusts. However, this ranking was not significant in Greece (*p* > 0.05), while in Ireland it was significant (*p* < 0.001). Post hoc pairwise comparisons with Bonferroni correction in Ireland showed significant differences between migratory locusts and yellow mealworms (*p* = 0.010) and between house crickets and yellow mealworms (*p* = 0.014).

The ranking of the other insect-based foods was significantly different in Greece and Ireland (*p* < 0.001, respectively). In both countries, participants were most willing to consume chips or baked goods made with insect flour, ground insects in herb/spice mixes, and granola/breakfast cereals, though the exact order varied slightly (see Table 4). Post hoc pairwise comparisons revealed no significant differences (*p* > 0.05) in willingness to consume foods where insects were not visible. However, participants were significantly less willing (*p* < 0.05) to consume foods where insects were either whole or visible, such as seasoned visible insects (roasted/fried), chocolate coated insects, and visible insects in burger patties compared to the other products (see Table 4).

When participants were asked to name other foods, they would most likely consume if they contained insects or insect-derived ingredients, 7% of participants in Greece and 21% in Ireland responded to this question. However, only a seventh of responders in Greece and half of the responders in Ireland provided actual food examples, while the remainder expressed unwillingness to consume any insect-based foods. The additional foods mentioned were typically those where insects would not be visible, such as biscuits, smoothies, cakes, sausages, and ingredients such as insect flour.

The percentage of insect protein participants were willing to consume in food differed significantly (*p* < 0.001) between those in Greece and Ireland (see Table 5). In Greece, 43% of participants indicated they would consume 0% insect protein compared to 22% in Ireland. The next most common response in Greece was 1–10% insect protein (17% of participants), while, in Ireland, 17% of participants were willing to consume 21–30% insect protein. Approximately 7% of participants in both countries were open to consuming foods in which 100% of the protein was derived from insects (see Table 5).

### 3.3. Reasons for (Un)Willingness to Consume Insect-Based Foods

The level of agreement with statements regarding reasons for willingness and unwillingness to consume insect-based foods differed significantly between participants in Greece and Ireland (see Table 6). Approximately twice as many participants in Ireland agreed with the reasons for willingness compared to those in Greece. However, in both countries, more participants expressed willingness to consume insect-based foods if they had pleasant sensory properties (34% in Greece and 68% in Ireland), were safe (34% in Greece and 67% in Ireland), and nutritious (30% in Greece and 60% in Ireland), compared to other reasons (see Table 6). The fewest participants in Greece (14%) were willing to consume insect-based foods for environmental reasons, while in Ireland (41%), portion size was the least motivating factor.

The degree of agreement with all the provided reasons for willingness to consume insect-based foods significantly and positively correlated (*p* < 0.001) with participants’ willingness to consume the various insect-based foods explored in this study, as well as the amount of insect protein they would be willing to consume in foods.

About 17% of participants in Greece and 38% in Ireland provided additional reasons for their willingness to consume insect-based foods. These reasons were categorised into two themes using thematic analysis: reasons related to the participants and reasons related to the insect-based foods. Table 7 presents the detailed themes, sub-themes, example codes, and frequencies for these additional reasons. Reasons related to the insect-based food were mentioned about six times more often by participants in both Greece and Ireland compared to reasons related to the participants. The most frequently mentioned reason in Greece was “if it is the only option” (n = 9), followed by “if it has specific health/nutrition benefits” (n = 8) and “if insects are not visible” (n = 5). In Ireland, the top reasons were “if insects are not visible” (n = 17), “if it has pleasant sensory properties” (n = 16) and “sustainability” (n = 11).

Table 6 shows that 82% of participants in Greece and 63% in Ireland indicated a lack of willingness to consume insect-based foods due to a preference for other foods. About twice as many participants in Greece found the concept disgusting or repulsive compared to those in Ireland. Nonetheless, over 50% of participants in both countries (64% in Greece and 55% in Ireland) agreed that they would be unwilling to consume insect-based foods due to insufficient information about the benefits and risks associated with them (see Table 6).

Agreement with the statements “I prefer to eat other foods” and ”I think it is disgusting/repulsive” significantly and negatively correlated (*p* < 0.01) with participants’ willingness to consume all insect-based foods examined, as well as the amount of insect protein they would be willing to consume. In addition, the agreement with the reason ”the thought of eating insects reminds me of something disturbing that I have seen” significantly and negatively correlated (*p* < 0.01) with participants’ willingness to consume most insect-based food in Greece, except for yellow mealworms, seasoned visible insects (roasted/fried), visible insects in burger patties, chocolate coated insects and ground insects in granola/breakfast cereals. In Ireland, this reason negatively correlated (*p* < 0.01) with willingness to consume all insect-based foods explored. In both countries, the lack of sufficient information about the benefits of insect-based foods had no significant effect (*p* > 0.05) on participants’ willingness to consume them.

A small percentage of participants (13% in Greece and 36% in Ireland) provided other reasons for their unwillingness to consume insect-based foods. Similar to the willingness reasons, thematic analysis identified two key themes: reasons related to the participants and reasons related to the insect-based foods (see Table 8). Reasons related to the insect-based foods were mentioned twice as often as those related to the participants. In Greece, psychological barriers (n = 8) and perceptions of insects as not beneficial (n = 7) were the most frequently cited reasons. In Ireland, the most frequently mentioned reasons for unwillingness were safety concerns (n = 19) and unpleasant sensory properties (n = 19) (see Table 8).

### 3.4. External Influence on Willingness to Consume Insect-Based Foods

The influence of external sources on willingness to consume insect-based foods differed significantly (*p* < 0.001) between participants in Greece and Ireland (see Table S3). Participants in Ireland were more likely to be influenced by external sources compared to those in Greece. Over 50% of participants in Greece indicated that social media (62%), television programmes/radio/news articles (62%), and a chef giving a live demonstration (51%) would “definitely not” influence their willingness to try insect-based foods. Similarly, less than 30% of participants in Greece may be influenced by any of the external sources explored (see Table S3). In contrast, over half of the participants in Ireland (52%) indicated that a live cooking demonstration by a chef could influence their decision, and 43% reported that family members might sway their willingness to consume insect-based foods.

Table 9 ranks the external sources by their influence on participants’ willingness to consume insect-based foods. For participants in Greece, family members would be the most influential, followed by peers/friends, with television programs/radio/news articles being the least influential. In Ireland, a live demonstration by a chef would be most influential, followed by family members, while social media would have the least influence. Friedman’s ranking of these external sources showed statistically significant differences in both countries (*p* < 0.001). However, post hoc pair-wise comparisons revealed no significant differences (*p* > 0.05) between social media and television programmes, or between peers and family members, or a chef’s live demonstration in either country. Significant differences (*p* < 0.001) were found between social media or television programs and peers/friends, family members, and a chef’s live demonstration.

### 3.5. Willingness to Buy Insect-Based Foods

The level of agreement with statements regarding the willingness to buy insect-based foods differed significantly (*p* < 0.001) between participants in Greece and Ireland (see Table 10). Overall, participants in Ireland were more willing to buy insect-based foods compared to those in Greece. Approximately 39% of participants in Greece and 20% in Ireland agreed with the statement, “I would never buy insect-based foods under any circumstances”. However, some of the participants indicated that they would consider buying insect-based foods under specific conditions. Most would be willing to buy them after trying a free sample and enjoying the sensory experience (31% in Greece and 59% in Ireland), or if the product closely resembled foods they typically buy (32% in Greece and 50% in Ireland). Only 6% of participants in Greece and 20% of participants in Ireland would be willing to buy insect-based foods without having sampled them beforehand. Similarly, a small percentage of participants (2% in Greece and 7% in Ireland) would buy insect-based foods if they were more expensive than the alternatives (see Table 10).

## 4. Discussion

This study compared willingness to consume insect-based foods between participants in Greece and Ireland, exploring the reasons behind their willingness or unwillingness, the potential to influence their decisions and their willingness to buy such foods. Overall, participants in Greece were significantly less willing to consume insect-based foods than their counterparts in Ireland. Comparing the EU-approved insect species investigated in this study, participants from both countries showed the highest willingness to consume yellow mealworms, followed by house crickets and migratory locusts. Similarly, in a focus group study with participants in Ireland, migratory locusts were also the least preferred [15]. In a study conducted among generation Z consumers in Greece, “deep-fried crickets” were more preferred than “mealworms” while “crickets” were less preferred than “mealworms” [34]. However, both of these studies [15,34] did not report whether these preferences were statistically significant or not. In the current study, the willingness to consume these insects (yellow mealworms, house crickets, and migratory locusts) was not statistically significant among participants in Greece. This is likely because over 75% of them were “extremely unwilling” to consume any of these insect species. In contrast, participants in Ireland were significantly more willing to consume yellow mealworms compared to the other species. Fischer and Steenbekkers [55] found that Dutch consumers preferred those insect species that they were already exposed to on the market. Even in countries with a traditional entomophagy culture such as Thailand, consumers would be more willing to consume the insect species they are more familiar with in terms of previous consumption while rejecting the ones they are not familiar with [16]. Yellow mealworms were the first insect species to be approved as novel food in the EU and therefore have been in the market longer, and possibly, participants have been exposed to them for longer. However, more research is needed to test this hypothesis.

Participants in both countries were less willing to consume insect-based foods when the insects were either whole or visible compared to foods where the insects were not visible. This finding aligns with previous research [15,18,37,40,56,57,58,59]. However, in the current study, willingness to consume the different foods where insects were not visible was not significantly different for participants in both countries. This suggests that participants are generally willing to consume insect-based foods where the insects are not visible regardless of the product. The slight (non-significant) differences regarding willingness to consume these products could be attributed to personal preferences [15].

Participants’ food choice motives, as seen in Table 2, reflected how they were influenced to consume insect-based foods. Participants in both Greece and Ireland were more inclined to consume insect-based foods if they were safe, nutritious, and had pleasant sensory characteristics. Likewise, health and pleasurable sensory sensations were the top two most important food choice motives for all participants. In addition, the environmental factor was the least important food choice motive for participants in Greece and their least motivating factor to consume insect-based foods. Similarly, past studies have linked participants’ food choice motives with factors affecting their willingness to consume insect-based foods [15,40].

Qualitative analysis in the current study revealed interesting findings wherein sustainability reasons were mentioned as a positive factor by participants in Ireland and as a negative factor by participants in Greece. Some participants in Ireland mentioned that they would be willing to consume insect-based foods for the good of the environment, while some in Greece were unwilling to consume insect-based foods because they were sure that these were “harmful to the environment” and “disruptive to the food chain”. The environmental sustainability potential of insects as an alternative protein food source has been investigated and confirmed by several studies [6,9,10]. However, consumers may not be aware of the environmental impact of foods [60,61] and providing information on the environmental benefits of insects as food has been found to increase acceptance [62]. Participants in Ireland may have had the knowledge of the sustainability potential of insects while those in Greece may have not. This underscores the need for educational interventions on the environmental benefits of entomophagy. Indeed, a recent study by Gkinali et al. [35] found that even a brief educational intervention on the benefits of entomophagy increased the willingness to consume insect-based foods among Greek consumers classified as “innovators”, “early adopters” and “early majority” according to Rogers theory [63].

Family members, peers/friends, a chef, and social media and television programmes would influence participants in Ireland more than those in Greece to consume insect-based foods. However, in both countries, media, such as television or social networks, would be the least influential. Negative portrayals of insects in the media [64] have been found to negatively affect willingness to consume insects [15], which may have contributed to media being the least influential in the current study. Participants in Greece would be most influenced by family members, while those in Ireland would be most influenced by chefs’ live demonstrations. This is consistent with findings from a recent review showing that different sources of external influence can have varied impacts on insect-based food acceptance [13].

Increasing consumption of insect-containing foods would increase demand and potentially enable automation of production processes and potentially lead to reduced costs [65,66]. This is crucial because more people would buy them if they were cheaper or less expensive than alternative foods without insects. Similarly, Giotis and Drichoutis [36] found that the majority of the Greek participants in their study were unwilling to buy insect-based foods when they were more expensive.

The sample sizes (283 participants in Greece and 206 participants in Ireland) used in the current study make the findings ungeneralisable to the entire population of consumers in these countries. We therefore recommend that future studies use our findings as a baseline and expand them to more participants in Greece and Ireland. In addition, willingness to buy insect-based foods was investigated in broad terms and not specific to different insect-based foods. This was carried out so as not to overwhelm participants with lots of questions, which may have affected the quality of responses. Despite the limitations, this study presents nuances in willingness to buy insect-based foods, which can be explored further when assessing consumer intention to buy the different insect-based products. Furthermore, this is the first study to provide insights into the acceptance of insect-based foods when comparing participants in Greece and Ireland, which could contribute towards an understanding of entomophagy acceptance in Western cultures [1].

## 5. Conclusions

The aim of this study was to compare the acceptance of insect-based foods between participants in Greece and Ireland, two EU countries with different culinary traditions, where insects are not widely available in the market. Overall, participants from both countries were more willing to consume food products where insects were not visible. However, participants in Ireland were more willing to consume all the insect-based foods explored, and were more inclined to buy these foods, as well as more positively influenced by external factors than participants in Greece. Moreover, participants in Ireland preferred slightly higher percentages of insect-protein inclusion in foods. Overall, findings from this study suggest that consumers in both countries are unlikely to buy insect-based foods without tasting them first (previous consumption), or if they are more expensive than alternative products. In addition, availability in local stores and resemblance to foods usually purchased may be relevant factors that could affect consumers’ willingness to buy insect-based foods. This study highlights the value of a cross-cultural approach, revealing significant differences in food choice motives, food neophobia scores, and reasons for (un)willingness to consume insect-based foods between participants in Greece and Ireland. These findings underscore how varying cultural contexts shape perspectives on insect-based foods, providing insights that would not have emerged from studies conducted in isolation within each country. Moreover, since insect-based foods are not widely available in these countries and consumers can get them mostly online, the findings show that the food industry should tailor strategies to cultural context in each country. While these findings may not be generalised, we recommend that future research should investigate the impact of sustainability educational interventions and public tasting events on consumer willingness to purchase and consume insect-based foods in Greece. In addition, the effect of public chef demonstrations on insect-based food acceptance could be further explored in Ireland.

## Figures and Tables

**Table 1:** Characteristics of the participants (N = 489).

Profile	Participants				Significance (Chi-Square)
	Greece (n = 283)		Ireland (n = 206)		
	n	%	n	%	*p*-Value
Age:					
18–29	109	38.5	72	35.0	0.169
30–39	55	19.4	50	24.3	
40–49	74	26.1	40	19.4	
50–59	32	11.3	34	16.5	
60 and above	13	4.6	10	4.9	
Gender:					
Male	68	24.0	61	29.6	0.135
Female	209	73.9	139	67.5	
Other	1	0.4	4	1.9	
Prefer not to say	5	1.8	2	1.0	
Farming background:					
No	242	85.5	145	70.5	**<0.001**
Yes	41	14.5	61	29.6	
Following a specific diet					
No	243	85.9	171	83.0	0.387
Yes	40	14.1	35	17.0	
Heard of insect-based foods before					
No	26	9.2	18	8.7	0.864
Yes	257	90.8	188	91.3	
Consumed insect-based foods before ^1^					
No, never	194	75.5	93	49.5	**<0.001**
I am not sure	46	17.9	30	14.6	
Yes, once	13	5.1	37	18.0	
Yes, more than once	4	1.6	28	13.6	
Previous consumption experience ^2^					
Negative	3	17.6	6	9.2	0.101
Neutral	12	70.6	34	52.3	
Positive	2	11.8	25	38.5	

^1^ Based on the total number of participants who had heard of insect-based foods before (n = 257 for Greece and n = 188 for Ireland); ^2^ Based on the total number of participants who had consumed insect-based foods before (n = 17 for Greece and n = 65 for Ireland); In bold: statistically significant values.

**Table 2:** The ranking of the food choice motives by participants in Greece (n = 283) and Ireland (n = 206) in order of importance.

Food Choice Motive	Friedman Test ^1^				Mann-Whitney *p*-Value ^3^
	Greece		Ireland		
	Mean Rank	Ranking ^2^	Mean Rank	Ranking ^2^	
It is healthy	2.72	1	2.57	1	0.260
It provides me with pleasurable sensations (e.g., texture, appearance, smell and taste)	2.84	2	2.93	2	0.375
It is affordable	4.67	4	3.70	3	**<0.001**
It is convenient (in buying and preparing)	4.12	3	4.08	4	0.776
Is it familiar	5.18	5	5.29	5	0.550
It is a mixture of different ingredients	6.26	7	5.58	6	**<0.001**
It is environmentally friendly	6.86	9	6.07	7	**<0.001**
It fits in with my culture	6.36	8	7.38	8	**<0.001**
It is like the one I ate when I was a child	5.99	6	7.40	9	**<0.001**

^1^ Ranking significance within each country: χ^2^(8) = 702.024, *p* < 0.001 for Greece and χ^2^(8) = 706.394, *p* < 0.001 for Ireland; ^2^ Ranked from most important food choice motive (1) to least important (9) within each country; ^3^ Significance in the importance of the different food choice motives when comparing the two countries; In bold: statistically significant values.

**Table 3:** Willingness to consume insect-based foods among participants in Greece (n = 283) and Ireland (n = 206).

Insect-Based Food	Extremely Unwilling		Somewhat Unwilling		Neither Willing Nor Unwilling		Somewhat Willing		Extremely Willing		Significance (Mann–Whitney U) *p*-Value ^1^
	Greece	Ireland	Greece	Ireland	Greece	Ireland	Greece	Ireland	Greece	Ireland	
	%										
Yellow mealworms	76	36.9	8.5	27.2	9.2	9.2	6.0	22.8	0.4	3.9	<0.001
Migratory locusts	78.8	44.2	8.5	26.7	7.1	9.7	4.6	15.5	1.1	3.9	<0.001
House crickets	75.6	45.6	9.5	24.3	8.8	11.2	5.3	15.0	0.7	3.9	<0.001
Seasoned visible insects (roasted/fried)	66.4	32.5	14.8	22.3	10.2	6.3	7.8	30.6	0.7	8.3	<0.001
Visible insects in burger patties	77.1	45.6	15.1	29.6	6.5	12.1	1.1	12.1	0.4	0.5	<0.001
Chocolate coated	73.1	35.0	13.4	21.8	9.2	13.6	3.9	22.8	0.4	6.8	<0.001
Ground insects in granola/breakfast cereals	57.2	22.8	9.2	12.1	13.8	8.7	15.5	36.4	4.2	19.9	<0.001
Ground insects in an herb/spice mix	51.6	20.9	11.3	8.7	13.4	10.2	19.1	36.4	4.6	23.8	<0.001
Chips/baked products containing insect flour	51.1	20.9	10.1	9.2	16.5	6.8	17.3	37.4	5.0	25.7	<0.001
Pasta containing insect flour	52.3	19.9	11.8	11.2	14.7	4.9	16.5	39.8	4.7	24.3	<0.001
Ground insects in sauce	58.1	23.3	12.5	11.2	12.9	8.9	11.5	36.9	5.0	20.4	<0.001
Ground insects in burger patties	58.8	24.3	12.5	9.7	9.7	9.2	14.3	34.5	4.7	22.3	<0.001

^1^ All values were statistically significant.

**Table 4:** Ranking of the EU-approved insect species and insect-containing foods according to participants’ (Greece: n = 283; Ireland: n = 206) willingness to consume them.

Insect Species ^1^	Friedman Test			
	Greece		Ireland	
	Mean Rank	Ranking ^3^	Mean Rank	Ranking ^3^
Yellow mealworms	2.02	1	2.19	1
House crickets	2.01	2	1.91	2
Migratory locust	1.97	3	1.90	3
**Insect-based foods (other) ^2^**				
*Insects invisible*				
Chips/baked products containing insect flour	5.74	1	5.93	2
Ground insects in an herb/spice mix	5.68	2	5.80	3
Pasta containing insect flour	5.59	3	5.94	1
Ground insects in granola/breakfast cereals	5.41	4	5.41	6
Ground insects in burger patties	5.16	5	5.53	4
Ground insects in a sauce	5.11	6	5.48	5
*Whole/visible insects*				
Seasoned visible insects (roasted/fried)	4.44	7	4.24	7
Chocolate coated insects	4.11	8	3.84	8
Visible insects in burger patties	3.76	9	2.83	9

^1^ Ranking significance within each country: χ^2^(2) = 2.693, *p* = 0.260 for Greece and χ^2^(2) = 31.715, *p* < 0.001 for Ireland; ^2^ Ranking significance within each country: χ^2^(8) = 422.215, *p* < 0.001 for Greece and χ^2^(8) = 483.198, *p* < 0.001 for Ireland; ^3^ Ranked from the one participants are most willing to consume (1) to the one they are least willing to consume (3 for different species and 9 for the other insect-based foods).

**Table 5:** The percentage of insect protein that participants would be willing to consume in a product.

**Percentage of Insect-Protein Present in a Product**	**Percentage of Participants Willing to Consume the Insect-Based Food**				**Significance** **Mann-Whitney**
	**Greece (n = 283)**		**Ireland (n = 206)**		
	**n**	**%**	**n**	**%**	***p*-Value**
0%	121	42.8	45	21.8	<0.001
1–10%	48	17.0	14	6.8	
11–20%	25	8.8	27	13.1	
21–30%	23	8.1	34	16.5	
31–40%	10	3.5	19	9.2	
41–50%	19	6.7	28	13.6	
51–80%	16	5.7	24	11.7	
81–100%	21	7.4	15	7.3	

**Table 6:** Participants’ (Greece: n = 283; Ireland: n = 206) level of agreement to the provided reasons for (un)willingness to consume insect-based foods.

I Am Willing to Eat Insect-Based Foods	Strongly Disagree		Somewhat Disagree		Neither Agree Nor Disagree		Somewhat Agree		Strongly Agree		Significance (Mann–Whitney U) *p*-Value ^1^
	Greece	Ireland	Greece	Ireland	Greece	Ireland	Greece	Ireland	Greece	Ireland	
	%										
If it closely resembles the food that I usually eat	50.5	19.9	11.7	13.1	13.8	14.1	20.8	39.8	3.2	13.1	<0.001
If it can be easily paired with other foods	55.1	21.4	15.9	10.2	12.4	12.1	14.1	41.7	2.5	14.6	<0.001
If it is good for the environment	52.7	22.3	13.8	14.6	19.4	19.4	9.2	31.6	4.9	12.1	<0.001
If it is safe to eat	43.5	17.5	10.6	7.3	11.7	8.3	17.3	26.2	17.0	40.8	<0.001
If I do not have to eat a bigger portion of t compared to the alternatives for the same nutritional benefits	50.2	22.8	15.8	13.1	18.6	23.3	12.2	29.1	3.2	11.7	<0.001
If it provides me with pleasant sensory experiences (e.g., texture, taste, appearance and smell)	43.0	19.9	8.6	4.4	14.3	7.3	19.4	35.9	14.7	32.5	<0.001
If it is nutritious	46.6	20.4	10.8	6.8	15.8	13.1	18.3	40.8	8.6	18.9	<0.001
**I would NOT be willing to eat insect-based foods because…**											
I prefer to eat other foods	3.9	2.9	4.6	12.6	9.2	21.8	27.9	28.2	54.4	34.5	<0.001
I think it is disgusting/repulsive	1.8	15.0	6.7	22.8	7.8	16.5	21.2	27.2	62.5	18.4	<0.001
I do not have enough information regarding the benefits/risks associated with it	8.5	7.8	8.5	14.1	18.7	22.8	29.3	34.0	35.0	21.4	0.005
The thought of eating insects reminds me of something disturbing that I have seen	33.9	17.0	19.1	20.4	23.3	22.3	8.8	24.8	14.8	15.5	<0.001

^1^ All values were statistically significant.

**Table 7:** Themes, sub-themes and example codes from the qualitative analysis of participants’ other willingness reasons to consume insect-based foods.

Theme	Sub-Theme	Example Codes ^1^	
		Greece (n = 47)	Ireland (n =79)
Participant related	Open-minded	Curiosity (3)	To try new things (4)
			As a one-time experience (1)
			As a challenge (1)
	Culture	If in China (1)	Already part of culture (2)
			Other cultures eat it (1)
			If it becomes normalised (1)
	Information	-	If given more information on reasons for consumption (1)
			If given more information on the number of people who already consume them traditionally (1)
Insect-based food related	Health/nutrition benefits	If it is healthy (1)	If it is healthy (3)
		If low in calories (2)	If nutritious (2)
		If low in fat (1)	High in protein (1)
		If beneficial to the immune system (1)	If better source of protein than meat (1)
		If beneficial to the body (1)	If high in glutamic acid (1)
		For therapeutic reasons (1)	As medicine (1)
		If recommended by a doctor (1)	If recommended by a doctor (1)
	Sensory properties	If tasty (1) If texture is same as conventional foods (1)	If it is tasty (7)
			If appearance is appealing (3)
			If texture is good (1)
			If ‘enjoyable’ (1)
			If sensory properties (appearance, texture and taste) are similar with conventional foods (4)
	Availability	If it is the only option (9)	If it is the only option (3)
	Affordability	If it is economical (1)	If it is cheaper than alternative foods (8)
	Insect visibility	If the insects are not visible (3) If ground up (2)	If insects are not visible (8)
			If ground up (5)
			As an ingredient in other foods (3)
			If in a paste (1)
	Sustainability	-	For the environment (8)
			Sustainability in general (3)
	Convenience	-	If it is ready-to-eat (2)
	Source	-	If locally produced (1)
			If ethically produced (2)
			If it is organic (1)
			If it is from a reliable source (1)
			If a person preparing is known to be a good cook (1)
	Type of insects	-	If the insects are small (1)
			If the insects are dried (1)
	Safe	If it is safe to eat (1)	If it is safe to eat (2)
	Indirect	-	In the form of animal products from animals that have been fed insects (1)
	Food label	-	If it specifies on the food label that it is organic (1)

^1^ The number of times the codes are mentioned is provided in brackets after each code.

**Table 8:** Themes, sub-themes and example codes from the qualitative analysis of participants’ other unwillingness reasons to consume insect-based foods.

Theme	Sub-Theme	Example Codes ^1^	
		Greece (n = 38)	Ireland (n = 75)
Participant related	Diet	Being a vegan/vegetarian (4)	Being a vegan/vegetarian (11)
			Preference to a Mediterranean diet (1)
			For religious reasons (1)
	Psychological barrier	Disgust (7)	Disgust (8)
		Picky eater (1)	Food neophobia (8)
			The whole concept (1)
	Culture	Not aligned with own culture (2)	Not aligning with own culture (4)
			If not socially acceptable (2)
Insect-based food related	Safety	If not safe to eat (2)	Safety concern (11)
		Not suitable for consumption (2)	Presence of allergens (4)
			Due to a medical condition (2)
			Unhealthy (1)
			Not suitable for humans (1)
	Sensory properties	Different texture (2)	Unfamiliar with taste (2)
		Unpleasant appearance (2)	Possibility of an unpleasant texture (4)
			Unpleasant appearance (8)
			Unfamiliar with texture (3)
			If it has a strong flavour (1)
			Not resembling meat (1)
	Insect visibility	-	If visible/unprocessed (8)
	Price	Not economical (1)	Not economical/if expensive (2)
	Lack of Benefits	If not beneficial (2)	Not beneficial (1)
		No reason to eat (7)	If less beneficial than alternatives (3)
			No need for it (2)
			Not a solution (2)
	Insect farming	Depends on what the insects feed on (1)	-
		Animal cruelty (1)	
	Availability	-	Not available in Ireland (1)
	Sustainability	Harmful to the environment (2)	-
		Disruptive to the food chain (1)	

^1^ The number of times the codes are mentioned is provided in brackets after each code.

**Table 9:** Ranking of external sources according to their influence on participants’ willingness to consume insect-based foods (Greece: n = 283; Ireland: n = 206).

Effect of Influence From	Friedman Test ^1^			
	Greece		Ireland	
	Mean Rank	Ranking ^2^	Mean Rank	Ranking ^2^
Family members	3.33	1	3.33	2
Peers/Friends	3.24	2	3.27	3
A chef giving a live demonstration on how to prepare insect-based foods	3.14	3	3.55	1
Social media (Twitter, Facebook, etc.)	2.66	4	2.25	5
Television programmes/radio/news articles	2.62	5	2.59	4

^1^ Ranking significance within each country: χ^2^(4) = 135.734, *p* < 0.001 for Greece and χ^2^(4) = 203.340, *p* < 0.001 for Ireland; ^2^ Ranked from the most influential source (1) to the least influential one (5) within each country.

**Table 10:** Participants’ (Greece: n = 283; Ireland: n = 206) willingness to buy insect-based foods.

I Would…	Strongly Disagree		Somewhat Disagree		Neither Agree Nor Disagree		Somewhat Agree		Strongly Agree		Significance (Mann–Whitney U) *p*-Value ^1^
	Greece	Ireland	Greece	Ireland	Greece	Ireland	Greece	Ireland	Greece	Ireland	
	%										
Never buy under any circumstances	24.7	37.4	21.6	28.6	14.8	14.1	6.0	4.9	32.9	15.0	<0.001
Only eat if it was offered to me free of charge	64.7	29.8	10.2	31.7	16.6	19.5	6.4	12.7	2.1	6.3	<0.001
Only buy after I have tried a free sample and enjoyed the experience (taste, texture, smell, etc.)	45.6	20.9	8.8	9.7	14.5	10.7	26.5	39.3	4.6	19.4	<0.001
Buy without having eaten it before	67.1	44.2	18.0	29.6	9.2	6.3	3.2	16.5	2.5	3.4	<0.001
Only buy if it was cheaper for the same amount/calories as the alternatives	54.4	20.4	13.8	16.5	17.7	22.8	12.7	29.6	1.4	10.7	<0.001
Buy if it is more expensive for the same amount/calories as the alternatives	73.5	55.8	18.0	27.7	6.4	9.2	1.8	5.8	0.4	1.5	<0.001
Buy if it costs the same price for the same amount/calories as the alternatives	59.4	30.6	14.8	19.4	15.5	22.8	8.5	20.4	1.8	6.8	<0.001
Only buy if it was available at local stores	55.1	21.4	12.0	13.1	18.7	21.4	11.7	35.4	2.5	8.7	<0.001
Only buy if it closely resembles the food that I usually buy	45.9	19.5	9.2	13.7	13.4	16.6	25.1	39.5	6.4	10.7	<0.001

^1^ All values were statistically significant.

## Data Availability

The data presented in this study are available on request from the corresponding author. The data are not publicly available due to ethical reasons.

## Supplementary Materials

The following supporting information can be downloaded at: https://www.mdpi.com/article/10.3390/foods14030490/s1, Table S1: Survey questions used in the present study; Table S2: The extent to which participants agreed to the Food Neophobia scale items (Mean ± SD) within each country and comparisons between countries; Table S3: The extent to which participants (Greece: n = 283; Ireland: n = 206) would be influenced by external sources to consume insect-based foods.
