# Supplementary material for: Cross-Cultural Perspectives on Insect-Based Foods: Insights from Consumers in Greece and Ireland
Source: Foods. 2025 Feb 3;14(3):490. doi: 10.3390/foods14030490 (PMC11816475; doi:10.3390/foods14030490)
Supplement: Supplementary file 1 [file foods-14-00490-s001.zip › foods-3431506-supplementary.pdf]

# Supplementary Material

**Table S1:** Survey questions used in the present study.

| | |
|---|---|
| First section | |
| 1) Which of the following do you view as important regarding the food you eat on a typical day? (Drag the most important attribute to the top and so on, so that 1 will correspond to the most important and 9 to the least important one) | It is a mixture of different ingredients |
| | It provides me with pleasurable sensations (e.g., texture, appearance, smell and taste) |
| | It is healthy |
| | It fits in with my culture |
| | It is affordable |
| | It is like the one I ate when I was a child |
| | It is convenient (in buying and preparing) |
| | It is familiar |
| | It is environmentally friendly |
| 2) To what extent would you agree/disagree with the following statements?<br>o I am constantly sampling new and different foods<br>o I don't trust new foods<br>o If I don't know what a food is, I wont try it<br>o I like foods from different cultures<br>o At dinner parties, I will try new foods<br>o I am afraid to eat things I have never seen before<br>o I will eat almost anything | Strongly disagree |
| | Somewhat disagree |
| | Neither agree nor disagree |
| | Somewhat agree |
| | Strongly agree |
| 3) Have you ever heard of the eating of insects or foods containing insects before? | No |
| | Yes |
| 4) Have you ever eaten insects or foods containing insects before?<br>*Question displayed only if 'Yes' is selected for Q3* | No, never |
| | I am not sure |
| | Yes, once |
| | Yes, more than once |
| 5) How was your experience of eating insects or foods containing insects?<br>*Question displayed only if 'Yes, once' or 'Yes, more than once' is selected for Q4* | Negative |
| | Neutral |
| | Positive |
| Second section | |
| 6) To what extent would you be willing/unwilling to eat the insect species shown below?<br>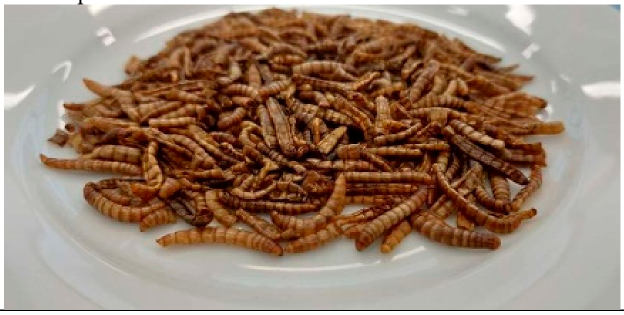 | Extremely unwilling |
| | Somewhat unwilling |
| | Neither willing nor unwilling |
| | Somewhat willing |
| | Extremely willing |
| 7) To what extent would you be willing/unwilling to eat the insect species shown below? | Extremely unwilling |
| | Somewhat unwilling |
| | Neither willing nor unwilling |
| | Somewhat willing |
| | Extremely willing |

| | |
|---|---|
| 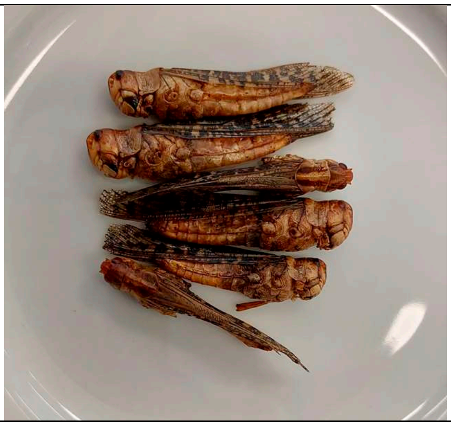 | |
| 8) To what extent would you be willing/unwilling to eat the insect species shown below?<br>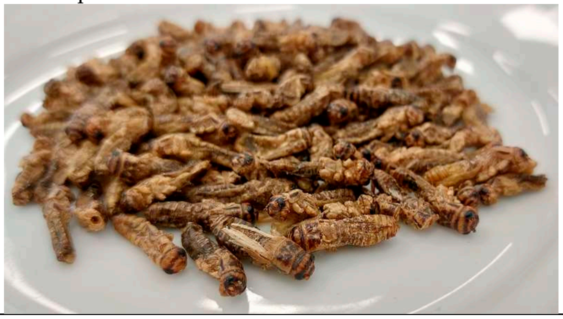 | Extremely unwilling<br>Somewhat unwilling<br>Neither willing nor unwilling<br>Somewhat willing<br>Extremely willing |
| 9) To what extent would you agree/disagree with the following statements: I am willing to eat insect-based foods…<br>o …if it closely resembles the food that I usually eat<br>o …if it can be easily paired with other foods that I usually eat<br>o …if it is good for the environment<br>o …if it is safe to eat | Strongly disagree<br>Somewhat disagree<br>Neither agree nor disagree<br>Somewhat agree<br>Strongly agree |
| 10) To what extent would you agree/disagree with the following statements: I am willing to eat insect-based foods…<br>o …if I do not have to eat a bigger portion of it compared to the alternatives for the same nutritional benefits<br>o …if it provides me with pleasant sensory experiences (e.g., texture, taste, appearance and smell)<br>o …if it is nutritious | Strongly disagree<br>Somewhat disagree<br>Neither agree nor disagree<br>Somewhat agree<br>Strongly agree |
| 11) If you have any other reasons for which you would be willing to eat insect-based foods, please state them below: | [Open-ended] |
| 12) To what extent would you agree/disagree with the following statements: I am NOT willing to eat insect-based foods because…<br>o …I prefer to eat other foods<br>o …I think it is disgusting/repulsive<br>o …I do not have enough information regarding the benefits/risks associated with it<br>o …the thought of eating insects reminds me of something disturbing that I have seen | Strongly disagree<br>Somewhat disagree<br>Neither agree nor disagree<br>Somewhat agree<br>Strongly agree |
| 13) If you have any other reasons for which you would NOT be willing to eat insect-based foods, please state them below: | [Open-ended] |
| 14) Insects can either be consumed as they are or they can be prepared and incorporated as ingredients in other foods. To what extent would you be willing/unwilling to eat the following insect-based foods? | Extremely unwilling<br>Somewhat unwilling<br>Neither willing nor unwilling<br>Somewhat willing |

| | |
|---|---|
| o Seasoned visible insects that have been fried/roasted<br>o Chocolate coated insects<br>o Ground insects in granola/breakfast cereals<br>o Ground insects in an herb mix/spice mix | Extremely willing |
| 15) To what extent would you be willing/unwilling to eat the following insect-based foods?<br>o Chips/baked products containing insect flour<br>o Pasta containing insect flour<br>o Ground insects in a sauce<br>o Ground insects in burger patties<br>o Visible insects in burger patties | Extremely unwilling<br>Somewhat unwilling<br>Neither willing nor unwilling<br>Somewhat willing<br>Extremely willing |
| 16) If there is any other food you would most likely eat if it contained insects/insect-derived ingredients, please state it below | [Open-ended] |
| 17) Insects are generally used as ingredients in a number of foods because of their high protein content. If you were to consume a high protein product containing insects, how much protein would you like to be replaced by insect protein? | 0%<br>1-10%<br>11-20%<br>21-30%<br>31-40%<br>41-50%<br>51-80%<br>81-100% |
| Third Section | |
| 18) To what extent would you agree/disagree with the following statements:<br>I would <u>ONLY</u>…<br>o …eat insect-based food if it was offered to me free of charge<br>o …buy insect-based foods after I have tried a free sample and enjoyed the experience (taste, texture, smell, etc)<br>o …buy insect-based food if it was cheaper for the same amount/calories as the alternatives<br>o …buy insect-based food if it was available at local stores<br>o …buy insect-based foods if it closely resembles the food that I usually buy | Strongly disagree<br>Somewhat disagree<br>Neither agree nor disagree<br>Somewhat agree<br>Strongly agree |
| 19) To what extent would you agree/disagree with the following statements:<br>o I would buy insect-based foods without having eaten it before<br>o I would buy insect-based foods if it is more expensive for the same amount/calories as the alternatives<br>o I would buy insect-based foods if it costs the same price for the same amount/calories as the alternatives<br>o Select 'somewhat disagree' as proof that you are reading the text<br>o I would never buy insect-based foods under any circumstances | Strongly disagree<br>Somewhat disagree<br>Neither agree nor disagree<br>Somewhat agree<br>Strongly agree |
| 20) To what extent would the following influence you to eat insect-based food?<br>o Peers/Friends<br>o Family members<br>o Social media (Twitter, Facebook, etc)<br>o Television programs/radio/news articles<br>o A chef giving a live demonstration on how to prepare such foods | Definitely will not<br>Probably will not<br>Might or might not<br>Probably will<br>Definitely will |
| Fourth section | |
| 21) How old are you? | 18-29 |

| | |
|---|---|
| | 30-39 |
| | 40-49 |
| | 50-59 |
| | 60 and above |
| 22) Which gender best describes you? | Male |
| | Female |
| | Other |
| | Prefer not to say |
| 23) Do you come from a farming background? | No |
| | Yes |
| 24) Do you follow a specific diet (vegetarian, vegan, calorie restricted, other)? | No |
| | Yes |
| 25) Could you please specify the type of diet that you follow? *Question displayed only if 'Yes' is selected for Q24* | [Open-ended] |

**Table S2.** The extent to which participants agreed to the Food Neophobia scale items (Mean ± SD) within each country and comparisons between countries.

| Food Neophobia Scale Item[1] | Greece | Ireland | Significance (Mann-Whitney) |
|---|---|---|---|
| | Mean ± SD | Mean ± SD | P-values |
| I am constantly sampling new and different foods | 3.59 ± 1.046 | 3.53 ± 1.176 | 0.776 |
| I don't trust new foods | 2.37 ± 1.038 | 2.00 ± 1.041 | **<0.001** |
| If I don't know what a food is, I won't try it | 2.52 ± 1.284 | 2.70 ± 1.396 | 0.231 |
| I like foods from different cultures | 3.93 ± 1.037 | 4.38 ± 0.852 | **<0.001** |
| At dinner parties, I will try new foods | 3.93 ± 1.049 | 4.15 ± 1.003 | **0.008** |
| I am afraid to eat things I have never seen before | 2.56 ± 1.331 | 2.22 ± 1.205 | **0.007** |
| I will eat almost anything | 2.87 ± 1.415 | 3.40 ± 1.437 | **<0.001** |

[1] Adapted from (Pliner & Hobden, 1992); In bold: statistically significant values

**Table S3.** The extent to which participants (Greece: n = 283; Ireland: n = 206) would be influenced by external sources to consume insect-based foods

| Source of influence | Definitely will not | | Probably will not | | Might or might not | | Probably will | | Definitely will | | Significance (Mann-Whitney U) P- value[1] |
|---|---|---|---|---|---|---|---|---|---|---|---|
| | Greece | Ireland | Greece | Ireland | Greece | Ireland | Greece | Ireland | Greece | Ireland | |
| | | | | | % | | | | | | |
| Peers/Friends | 44.5 | 21.4 | 12.7 | 12.1 | 19.8 | 26.7 | 19.4 | 34.5 | 3.5 | 5.3 | <0.001 |
| Family members | 44.5 | 23.3 | 11.0 | 10.2 | 18.4 | 23.8 | 21.2 | 35.9 | 4.9 | 6.8 | <0.001 |
| Social media (Twitter, Facebook, etc) | 61.8 | 36.9 | 15.5 | 27.2 | 14.8 | 24.3 | 7.1 | 10.2 | 0.7 | 1.5 | <0.001 |
| Television programs/radio/news articles | 61.8 | 29.1 | 15.9 | 26.7 | 14.8 | 22.3 | 6.4 | 19.9 | 1.1 | 1.9 | <0.001 |
| A chef giving a live demonstration on how to prepare such foods | 50.5 | 20.4 | 12.0 | 10.2 | 17.0 | 17.0 | 17.3 | 41.7 | 3.2 | 10.7 | <0.001 |

[1] All values were statistically significant
